# Supplementary material for: Patterns of Intron Gain and Loss in Fungi
Source: PLoS Biol. 2004 Nov 30;2(12):e422. doi: 10.1371/journal.pbio.0020422 (PMC532390; doi:10.1371/journal.pbio.0020422)
Supplement: Table S1 — Also available at http://genes.mit.edu/NielsenEtAl/. (4.3 MB ZIP). [file pbio.0020422.st001.zip › NielsenEtAl/html/688.html]

AN5662.1.NCU02380.1.MG03551.1.FG01248.1


```
 CLUSTAL W (1.82) Multiple Sequence Alignments - Introns Inserted


Sequence 1: NCU02380.1	788 aa
Sequence 2: MG03551.1	812 aa
Sequence 3: FG01248.1	745 aa
Sequence 4: AN5662.1	722 aa
Alignment Length: 821 aa
Number Identitical Residues: 295 aa
Alignment Score (without introns) 16889


MG03551.1 	MEAAKETVEAVAEGVKNVAIGSSEGKKPAGEAKKGGAKKEKKPAAGSADTGPLE~LDPPP
NCU02380.1	---MEQVTEKVTETLKKVAIG---GDKPQKEKKQ---KKEKGAPAAGGDAGPLE~MNPPP
FG01248.1 	MSSDQADAAKAAEGATEQSLP----SRPAKQPKD---KQPKDKSAKGGKSSGLE0MAEPP
AN5662.1  	---------MASDGPKDLPVR------------------------AAAEAKPAS1GDTLP
          	          .::  .. .:                          ...:   .     *

MG03551.1 	SFLKDRLDLFERLRKEYDDEVAQ~KPREPITITMPDG----SIKNGTAWETTPADIAKGI
NCU02380.1	AFIQERLDLFDKLYKEQQEELAK~RPRDDILITMPDG----TTKTGKAYETTPAEIAKGI
FG01248.1 	QFIQHRLDIFDKIKARQDAEIAA1KPREDIVISLPNG----KEEKGTSWETTPGAVARNI
AN5662.1  	DFIIERNKLFEELWQEHLEEVKN~RPHPEINVTLQLGDGNTTTIPSKAWETTPAQLLKHV
          	 *: .* .:*:.:  .   *:   :*:  * :::  *...:.   ..::****. : : :

MG03551.1 	SNSLFKRTVVARLDGDKEQLWDLERPLERSCKLELLDFSDPQGQFVFWHSSAHILGEAAE
NCU02380.1	SNSLFKRTVVARIDG--ETLWDLERPLEKSCKLELLDFNDDQGKFVFWHSSAHILGEACE
FG01248.1 	SKSLFERTVISRVDG---ELWDLTRPLEKSCKVEFLDFDHTEGKKVFWHSSAHILGEAAE
AN5662.1  	PKEISADIVIAKVDG---QLWDLGRPLEGECTVSYVPFSDPEGREVFWHSSAHCLGEACE
          	.:.:    *::::**    **** **** .*.:. : *.. :*: ******** ****.*

MG03551.1 	RRFGCSLCIGPPVDN--GFYYEMALPDG~AAVQSSDWKPLETLVSKIVKEKQKFERLVLS
NCU02380.1	RRFGCSLCIGPPVDN--GFYYEMALPGG~AAVQSSDWAPLENIVTKIVKEKQPFQRLEMS
FG01248.1 	RRFGCYLCNGPPTEDPPGFYYDMANMGE2QSVTEEDKKALEQLSNSIVKQKQPFERLEMT
AN5662.1  	CEYGCMLSHGPPTPQ--GFFYDMAMPDN2RVVKESDWKTLDNRANKIFKEKQSFDRLEVT
          	 .:** *. ***. :  **:*:**  .    * ..*  .*:   ..*.*:** *:** ::

MG03551.1 	KEDLLEMFKYNKYKQHIIKDKIADGTKTTVYRNGPLIDLCRGPHVPDTGRIEAFAIMK~N
NCU02380.1	KEDLLKMFAYNPYKQHIIKDKIPDGTRTTVYRNGPLIDLCRGPHVPDTSRIEAFAIMK~N
FG01248.1 	KEELLEMFEYSEYKKYFIQQRVPDGTKSTVYRCGPLIDLCRGPHVPTTGNIKAFSVLK0N
AN5662.1  	KENLRKMFAYSKYKLHYI-DKLVTGEKSTVYRCGTLVDLCRGPHIQSTGKVKTFKIMQ0N
          	**:* :** *. ** : * :::  * ::**** *.*:*******:  *..:::* ::: *

MG03551.1 	SASYFLGDKDNDSLQRIYGVSFPTKKQLTEYQKFLEEAAKRDHRRIGKDQELFFFHDYSP
NCU02380.1	SSSYFLGDANNDSLQRIYGVSFPDKKQMAAHKKFLEEAAKRDHRLIGKQQELFYFEECSP
FG01248.1 	SAAYWLGDSKNESVQRIAGVSFPDKKALEEYKHFLAEAAKRNHRKIGQDQKLFFFDEASP
AN5662.1  	SSAYFLGDQNNDSLQRIRGVAFPDKKQMAEHLKFLEEAEKRNHLRIAKEQELFFFDDVSP
          	*::*:*** .*:*:*** **:** ** :  : :** ** **:*  *.::*:**:*.: **

MG03551.1 	GSAMWLPHGTRIYNTLLSYIREIYWEKNYEEV~ITPNIYNADLWKQSGHWQFYKDDMFVI
NCU02380.1	GSAMWLPHGMRINNAIMEYIKEEYWKRGYDEV~MTPNMFNVALWEQSGHLAHYKDDMFIL
FG01248.1 	GSAFFLPHGVRIYNALMDLIKGEYQKRDFEEV1MSPNMYKADLWKTSGHWGHYEENMFTF
AN5662.1  	GCPFLLPNGVRIFNALQSLLRSEYRKRGYQEV~QTPNMYDVGIWKTSGHWAHYKDDMFKL
          	*..: **:* ** *:: . ::  * ::.::**  :**::.. :*: ***  .*:::** :

MG03551.1 	KDEKDTFGLKPMNCPGHALMFAHRERSHRELPWRVADFGVLHRNEASGALSGLTRVRRFQ
NCU02380.1	DVDKEQFGLKPMNCPAHAMMFRHRDRSHKELPLRLADFGVLHRNEASGALSGLTRVRRFQ
FG01248.1 	EVEKEKFGLKPMNCPGHCKIFAHSDVTYKDLPWRMADFGTLHRNEFSGALSGLTRVRRFQ
AN5662.1  	DVEKREWALKPMNCPGHFVLFGHRERSYRELPLRIADFGVLHRNEASGALHGLTRVRKFQ
          	. :*  :.*******.*  :* * : ::::** *:****.***** **** ******:**

MG03551.1 	QDDAHIFCREDQ~IKDEILDLFDFLRKMYTVMGLTFKLRLSTRPEKYMGAIETWDKAEES
NCU02380.1	QDDAHIFCREDQ~IKAEVADLFDFMKSFYGMLGLTFKLKLSTRPEKYMGEIETWDRAEAR
FG01248.1 	QDDAHIFCTVDQ0IREEIESAFDFLSSVYGIFGFTFKLKLSTRPEKYVGDIATWDAAEKK
AN5662.1  	QDDTHIFCTHDQ0IESEIEGLFDFLQSIYGLFGFTFKLKLSTRPEKYLGELETWNYAEEQ
          	***:****  ** *. *: . ***: ..* ::*:****:********:* : **: **  

MG03551.1 	LRQALDEFAASEGGVAWELNAGDGAFYGP0KIDIAIMDCLNREWQCATIQLDFVQPQNFN
NCU02380.1	LKEALNEFVAATGG-TWELNEGDGAFYGP~KIDIAVLDCLNRPWQCATIQLDFQQPINFS
FG01248.1 	LEEALESFAEKTGA-KWEFNPGDGAFYGP~KIDIALFDALKREHQCGTMQLDFNLPRRFK
AN5662.1  	LKKAMTKFKG----DDWTIDEGDGAFYGP~KIDITIADALKREFQCATIQLDYQAPLNFK
          	*.:*: .*        * :: ******** ****:: *.*:*  **.*:***:  * .*.

MG03551.1 	LEYMAGEDAAETKAKAEGAKITAPKVKVKDPQAIIDSAAAARGETVVEPAGVEGDKKEKE
NCU02380.1	LEYMT----ADAQAKQDEDKAASKAAKPEAP-----------KEEEEAPKEGEEKKKKAL
FG01248.1 	LRYVAN----------KGETGVSDGSNPEED-----------------------------
AN5662.1  	LEYMTG----------EKTQATQEEAKKEAD-----------------------------
          	*.*::.          .    .    : :                               

MG03551.1 	KVIKKLSPGCARPVMIHRAMAGSIERFTGILCEHYAGKWPLWLSPRQILVVPVGKGFYDY
NCU02380.1	LVKKPLTAGCARPVMIHRAMAGSIERFTAILAEHFAGKWPFWMSPRQVMVIPVGMGFLDY
FG01248.1 	-----LPAGYARPVMIHRAVLGSFERMFGILTEHFGGKWPFWLSPRQVLVVPVMPAANDY
AN5662.1  	PKSNEPAPGRARPVVIHRAIIGSFERFLGIITEHFGGKWPFWISPRQVLIVPVMPAVNDY
          	   .  ..* ****:****: **:**: .*: **:.****:*:****::::**  .  **

MG03551.1 	AEEVRSIFKKAKMFVD~VDTSGN0TLMKKIRTGQLAQY~NFIFV1VGAEEMNGRQVNVRN
NCU02380.1	AKEVALTLKKEKIHVD1VDSSGN~TLQKKIRSAQLAQY1NFIFV~VGDDEKTNRKVNIRY
FG01248.1 	AQEVQKIFKAKGLYSD~VDLSSN~TFQKKIRKGQLEQF~NFIFV1VGAEEKESRTLNIRN
AN5662.1  	AREVQKILQNDKLYVD~VDVTGN~TLQKKIRTGQLAQY~NFILV~VGAEEKESRSVNIRN
          	*.**   ::   :. * ** :.* *: ****..** *: ***:* ** :*  .* :*:* 

MG03551.1 	RDDPATQDRGQPIALEEALEKLAKLRDDKGTYNPFPKAAAEKKETAPAA---
NCU02380.1	RDDTSTQARDVPYSLEEAVEKLRALKADRGMYNPFPAHNPKKEEATKEEVKA
FG01248.1 	RDDQSTQAKGELVAIDEALEKLLQLKDTRGLVNKL-----------------
AN5662.1  	RDDTSSQSRGVVVPLEEARQKFRALRKERRLLNTL-----------------
          	*** ::* :.   .::** :*:  *:  :   * :
```
